# Initial Sequencing and Characterization of the Gastrointestinal and Oral Microbiota in Urban Pakistani Adults

**DOI:** 10.3389/fcimb.2020.00409

**Published:** 2020-08-07

**Authors:** Maria Batool, Syed Baqir Ali, Ali Jaan, Kehkishan Khalid, Syeda Aba Ali, Kaynat Kamal, Afraz Ahmed Raja, Farzana Gul, Arshan Nasir

**Affiliations:** Department of Biosciences, COMSATS University Islamabad, Islamabad, Pakistan

**Keywords:** microbiome, 16S rRNA sequencing, Pakistan, gut micriobiome, oral microbiome

## Abstract

We report the initial characterization of the gastrointestinal tract (gut) and oral microbiota (bacteria) in 32 urban Pakistani adults. Study participants were between ages 18 and 40, had body mass index between 18 and 25 Kg/m^2^, and were students or early-career professionals. These individuals donated a total of 61 samples (32 gut and 29 oral) that were subjected to 16S ribosomal RNA (rRNA) gene sequencing. Microbiome composition of Pakistani individuals was compared against the uBiome database of selected individuals who self-reported to be in excellent health. We observed strong gender-based differences in the gut microbiome of Pakistani individuals, a skewness toward Firmicutes, and unusually high levels of Proteobacteria in the Pakistani men. These observations may indicate microbiota dysbiosis, though 16S data alone can neither establish cause nor effect to human health. Albeit conducted on a smaller scale, our report provides a first snapshot about the composition and diversity of gut and oral microbiota communities in Pakistani individuals.

## Introduction

Several nation-wide microbiome sequencing projects have been completed or are underway in the developed world. In turn, the composition and diversity of human microbiota in developing or low-income countries are relatively less understood. This knowledge gap limits the global application of emerging and promising microbiota-inspired treatments (e.g., fecal microbiota transplant; Aroniadis and Brandt, 2013; Conrad and Vlassov, 2015). With a population of >200 million, Pakistan is the fifth most populous country in the World. It is linguistically, ethnically, and geographically diverse. Pakistani food is likely a mixture of high-fat and high-starch diet and low in fiber (personal communication with local nutrition biologist). Fresh fruit intake is low [~50% in a recent survey (Khuwaja and Kadir, 2010)] and “junk food” consumption is increasing, especially in the urban areas (Yahya et al., 2013). Moreover, uncontrolled persistent consumption of antibiotics through adulthood is becoming a concern (Aziz et al., 2018). Combined with low physical activity [up to 60% in a recent survey (Khuwaja and Kadir, 2010)], these factors can influence the diversity and composition of the Pakistani microbiota.

Here, we present the results of the pilot phase of the Pakistan Microbiome Initiative, a project launched to characterize the gut and oral microbiome of Pakistani individuals. While conducted on a smaller scale, the study provides interesting initial insights that can help design larger and informed studies in the future. For example, we discovered strong gender-based differences between the microbial profiles of Pakistani males and females, and overrepresentations of Firmicutes and Proteobacteria in females and males, respectively. From our analysis of participant self-reported metadata (Table S1), we also learned about the generic dietary habits of Pakistani individuals. These findings collectively provide an initial snapshot about the Pakistani microbiome and add novel data from a relatively understudied population.

## Materials and Methods

### Participant Identification and Screening

**Figure 1:**
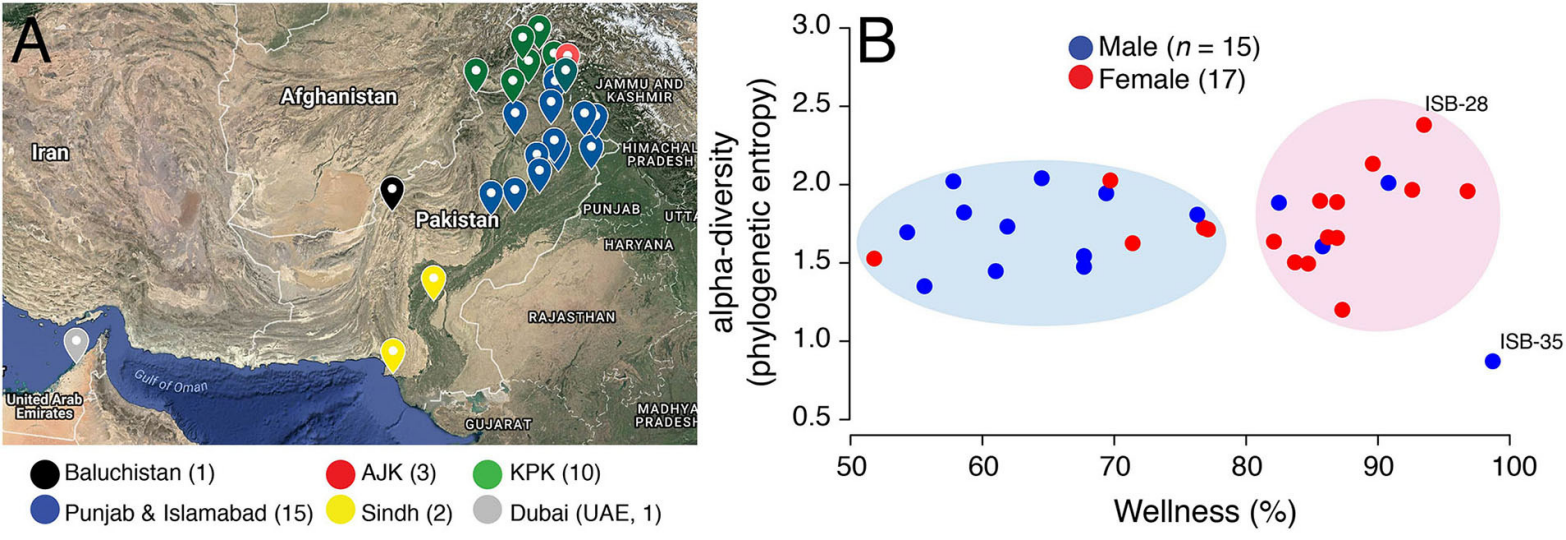
**(A)** Birth cities of study participants are highlighted on the map of Pakistan. Data points are colored according to geographical regions. Islamabad (Federal Capital) was pooled into Punjab for ease of visualization. Map generated online using Google Maps. AJK, Azad Jammu and Kashmir; KPK, Khyber Pakhtunkhwa. **(B)** A scatter-plot displaying the relationship between wellness (%) and alpha-diversity (phylogenetic entropy) for sampled Pakistani females (red) and males (blue). Individuals with maximum and minimum diversity scores are labeled (see Table S1 for metadata).

We recruited study participants through phone and in-person interviews conducted at the COMSATS University Islamabad between 2017 and 2018 (project sampling site). The major exclusion criteria were body mass index (BMI) either <18 or >25 Kg/m^2^, prior history of colon cancer, pregnant or lactating women, or women with irregular menstrual cycles (i.e., <21 or >35 days apart). We attempted to capture the maximum gender, geographic, and ethnic diversity of the country. Our shortlisted study participants therefore included 32 individuals (17 females and 15 males, mean age = 23.41, *SD* = 4.83 years) who donated a total of 61 stool and saliva samples and were born in 25 major cities (Figure 1A) representing seven major geographic regions (Punjab, Khyber Pakhtunkhwa (KPK), Baluchistan, Azad Jammu and Kashmir (AJK), Federal Capital Islamabad, Sindh, and United Arab Emirates (UAE) and six major ethnicities (Baloch, Kashmiri, Pashtun, Punjabi, Saraiki, and Sindhi) (Table S1). All individuals had “normal” BMI values ranging from 18.1 to 24.9 Kg/m^2^ (mean = 22.0, *SD* = 2.14 Kg/m^2^). Six individuals reported antibiotic intake in the 3 months prior to sampling, four individuals reported acute or chronic diarrhea in the 2 months prior to sampling, one individual had history of inflammatory bowel disease (IBD), five individuals had been diagnosed in the past with a medical condition (chronic liver disease followed by liver transplant, rheumatoid arthritis, hepatitis A, and tuberculosis), while one individual experienced constipation in the 2-month period prior to sampling. In addition, one individual reported international travel outside Pakistan (United Arab Emirates) in the 6-month period prior to sampling. This study was carried out in accordance with the recommendations of the Declaration of Helsinki. The protocol was approved by the COMSATS Ethics Review Board. All subjects gave **written informed consent**. Participant data was analyzed in aggregate and anonymously.

### Sample Collection

Stool and saliva samples were self-collected by participants at home in uBiome Explorer kits. These kits follow the protocols outlined by the NIH Human Microbiome Project (Keitel et al., 2010). To avoid overgrowth of some bacteria (especially *Gammaproteobacteria*) at room temperature (Amir et al., 2017), we specifically instructed and guided the participants to wash their hands and use sterile swabs/wipes to transfer small amounts of fecal material and saliva into vials containing lysis and stabilization buffer for DNA storage at room temperature. Samples were shipped to uBiome for further processing and sequencing.

### 16S Ribosomal RNA (rRNA) Gene Sequencing, DNA Extraction, and PCR Amplification

These steps were performed in the CLIA-compliant (Clinical Laboratory Improvement Amendments) and CAP-accredited (College of American Pathologists) uBiome laboratory in San Francisco, CA. Samples were lysed using mechanical bead-beating (Hummel and Kula, 1989). DNA was extracted and purified by a liquid-handling robot in a class 100 clean room using the method described in Cady et al. (2003). The V4 region of 16S rRNA gene was PCR amplified using universal forward and reverse primers. Primers also contained Illumina tags and barcodes with unique combination of forward and reverse indexes to allow multiplexing. Pooled PCR products were column-purified and selected through microfluidic DNA fractionation based on size (Minalla et al., 2001). Real-time qPCR quantified consolidated libraries using the Kapa Bio-Rad iCycler qPCR kit on a BioRad MyiQ prior to sequencing.

### DNA Sequencing and Quality Control

16S amplicons from each sample were individually barcoded and sequenced in multiplex in the NextSeq 500 platform in a 150bp paired-end modality. Raw data from the sequencer was first demultiplexed, and the forward and reverse reads obtained in each of the four lanes per sample were filtered using the following criteria: (i) Both forward and reverse reads in a pair must have an average Q-score > 30, and (ii) primers, and any leading random nucleotides (used to increase diversity of the library being sequenced) were trimmed, and forward reads were capped at 125 bp and reverse reads were capped at 124 bp. Forward and reverse reads of each pair were appended, and those sequences that contained more than 8 consecutive same nucleotide repeats were discarded. Remaining sequences were clustered using a distance of 1 nucleotide using the Swarm algorithm (Mahé et al., 2014) and the most abundant sequence per cluster was considered the representative of the cluster and assigned a count corresponding to the sum of sequences that composed the cluster. A chimera removal using these centroid representative sequences was performed using the VSEARCH uchime_denovo algorithm (Rognes et al., 2016). Singletons that remained after chimera removal were also discarded.

### Taxonomy Assignment

Quality-controlled forward and reverse reads that matched with >77% sequence identity to the same sequence in version 123 of the SILVA database [retrieved from https://www.arb-silva.de (Glöckner et al., 2017)] were assumed to be 16S sequences. The most abundant forward-reverse read pair per Swarm cluster was assigned taxonomic annotation according to the following thresholds: >97% identity (species), >95% (family), >85% (order), >80% (class), >77% (phylum). In total, we assigned taxonomy to ~5.5 million read-pairs (total = 5,580,520, 174,391/sample, minimum = 7,847, maximum = 353,529) in 32 gut samples and to ~4.3 million sequences (total = 4,380,874, 151,064/sample, minimum = 5,805, maximum = 386,012) in 29 oral samples. Two samples that were <10,000 read count threshold were dropped from the downstream analyses (Almonacid et al., 2017). These included a stool sample donated by ISB-35 and saliva sample donated by ISB-11. ISB-35 had recently undergone liver transplant and was taking immunosuppressants that likely reduced their gut microbiota diversity (Table S1). ISB-11, however, appeared normal, as per our questionnaire (Table S1) but had abnormally low-counts of oral bacteria relative to other samples. To ensure consistency in target taxa detection even at low abundances, this sample was also removed in the downstream analyses. Remaining samples (*n* = 59, >10,000 taxa) were rarefied to minimum library sizes (gut = 14,574/samples, oral = 11,878/sample) as there was still >10X difference between the library sizes of few small samples vs. the maximum library size (Weiss et al., 2017).

### Bioinformatics Analysis

MicrobiomeAnalyst (Dhariwal et al., 2017) was used to perform downstream analyses. A taxonomy abundance table containing features (phyla, genera, and species) and their abundance scores (detected raw counts at phyla, genera, and species levels) for all samples was provided as input along with sample metadata (Table S1). Low-count and low-variance features were removed, keeping only features with >4 count in at least 20% samples. Raw counts were rescaled using the method of total sum scaling. Weighted UniFrac distance was used to plot sample dissimilarity on the two main principal coordinates. Statistical significance of dissimilarity between study groups (e.g., gender) was evaluated by the Analysis of Similarities (ANOSIM) method implemented in MicrobiomeAnalyst. Within-sample diversity (i.e., alpha-diversity) was evaluated by abundance-weighted phylogenetic entropy index (McCoy and Matsen, 2013), which is linearly dependent on branch lengths in the phylogenetic tree and increases with the distinctiveness of the sample (Allen et al., 2009), along with traditional alpha-diversity measures (observed, Chao1, and Shannon's diversity). Linear Discriminant Analysis Effect Size (LEfSe) algorithm was implemented for biomarker discovery (Segata et al., 2011). Differential abundance of taxa was evaluated for key metadata collected from participants including gender, geography, ethnicity, dietary and social habits, and medical health questions (Table S1). Statistical significance was evaluated either by the two-tailed Wilcoxon rank sum test (2 samples) or Kruskal-Wallis (>2 samples), where appropriate (*P* < 0.05, *FDR* < 0.05). Abundance correlations were determined by Kendall rank correlation coefficient (τ) and visualized by heatmaps.

### uBiome Citizen Science Initiative

Relative abundances of major phyla and genera detected in the Pakistani gut and oral samples were compared against the gut and oral samples of self-reported healthy individuals in the uBiome Citizen Science initiative (Almonacid et al., 2017). These data were available from uBiome Explorer to all commercial users and to uBiome academic grant awardees for research purposes prior to uBiome closure. The uBiome healthy individuals completed a detailed questionnaire about 42 different medical conditions covering infectious diseases, metabolic disorders, chronic health issues, and mental health disorders and had never been diagnosed with abnormal blood glucose levels, diabetes, or digestive-tract related disorders or any other medical condition (see Almonacid et al., 2017 for details). Informed consent was obtained from all participants and these individuals consented to share their results for research purposes. This study was performed under a Human Subjects Protocol provided by an IRB (E&I Review Services, IRB Study #13044, 05/10/2013).

## Results

### Gender-Wise Differences in the Gut Microbiome of Pakistani Individuals

A wellness match (%), defined by uBiome Explorer, is the overlap in the microbiome composition between the samples of interest (Pakistani samples) and selected samples from individuals who reported no ailments (Almonacid et al., 2017). Wellness percentage in Pakistani individuals ranged from 51.80 to 98.70% (mean = 76.73%, SD = 13.55%) (Figure 1B). Interestingly, wellness percentage was significantly higher in females vs. males (mean = 82.51% vs. 70.17%, *P* = 0.01, two-tailed Wilcoxon rank sum test) suggesting that Pakistani women on average harbored a gut microbiome profile that was relatively more similar to the gut microbiome profiles in uBiome healthy individuals. In fact, Pakistani samples segregated into two major clouds when plotted against the wellness match and alpha-diversity [i.e., the number and distribution of taxa within a sample (McCoy and Matsen, 2013)], as measured by the phylogenetic entropy index (Allen et al., 2009; Figure 1B). The right cloud included samples with wellness match >80% and included 12/17 (71%) females. In comparison, the left cloud exhibited wellness match scores between 50–80% and predominantly included males (11/15, 73%). This result, however, has no bearing on health and does not imply that Pakistani women are healthier than Pakistani men. It simply means that there were gender-wise differences in the gut microbiome profiles of Pakistani individuals when compared with an ostensibly healthy Western cohort.

The phylogenetic entropy ranged from 0.873 to 2.381 with a mean of 1.727, *SD* = 0.29. While, numerically females had higher mean alpha-diversity compared to males (1.76 vs. 1.68), the difference was statistically insignificant (*P* = 0.62, two-tailed Wilcoxon rank sum test). The individual with the lowest diversity (ISB-35) had recently undergone liver transplant after chronic liver infection (Table S1). This individual was taking immunosuppressants that significantly reduced their gut microbiome diversity (removed from subsequent analyses). In comparison, individual with the maximum gut diversity (ISB-28) was interestingly the only one who reported no intake of “naan” (Table S1), a typical Pakistani/Indian bread made from wheat flour and served usually with ghee/oil. Naan/roti are rich in carbohydrates and a mandatory part of almost every Pakistani meal, especially in the Punjab province. This individual was therefore likely on “low-carb” diet relative to other sampled Pakistanis. Other than gender, however, wellness scores and diversity of samples did not differ significantly either by geography or ethnicity (Figure S1). Although, given the small sample size and high heterogeneity within ethnic and geographic divisions, these inferences lack robustness.

**Figure 2:**
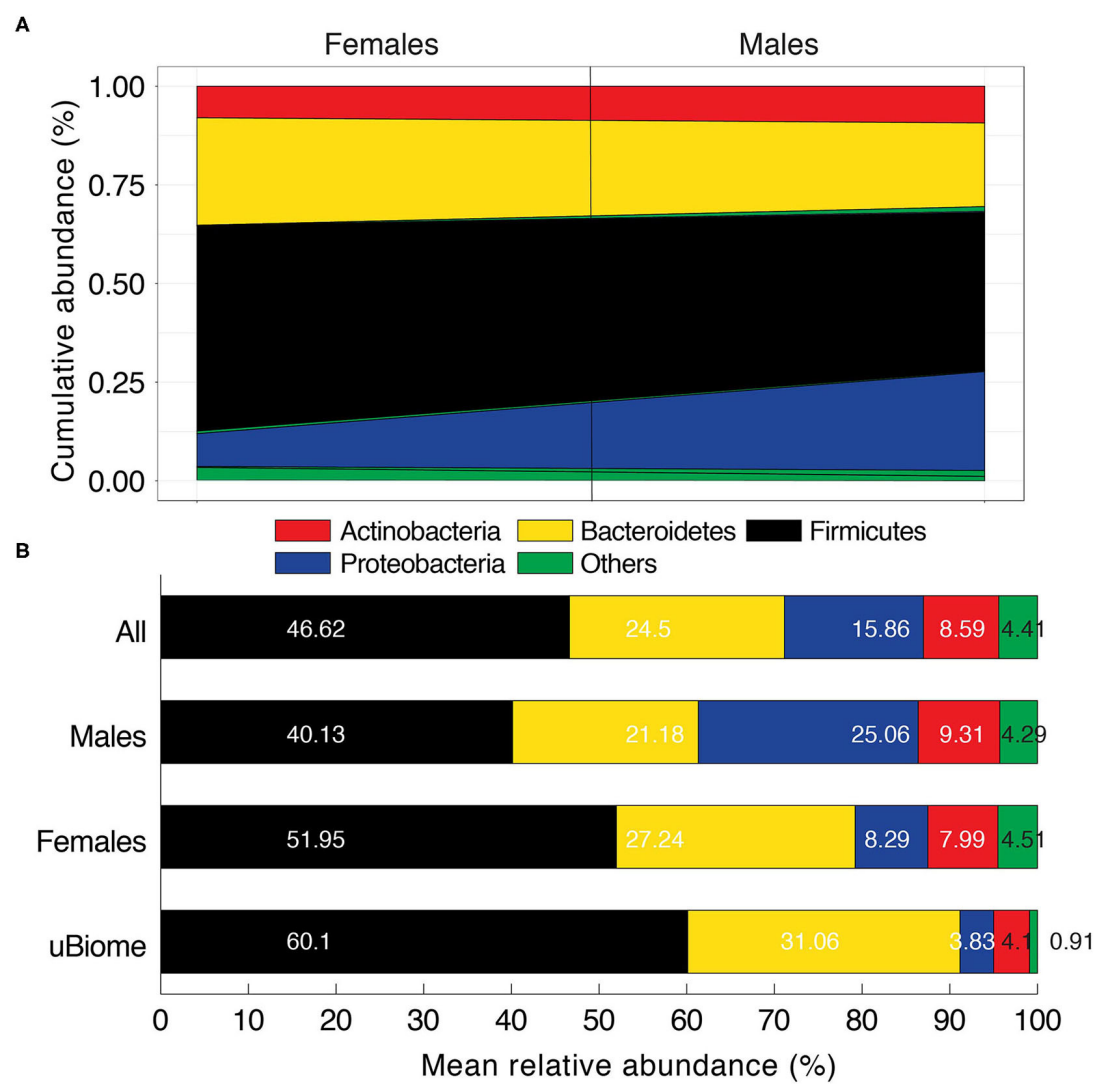
**(A)** Area diagram displaying the composition of microbial phyla detected in Pakistani gut samples. Black line separates male and female samples. *Others* category includes Tenericutes, Lentisphaerae, Euryarchaeota, Elusimicrobia, Verrucomicrobia, Fibrobacteres, Spirochaetes, and Synergistetes. **(B)** Comparison of mean relative abundance of microbial phyla for Pakistani males and females vs. uBiome dataset. Numbers on bars indicate actual percentages.

After the removal of ISB-35 and quality-control and filtering (see Materials and Methods), we determined that four major phyla, Firmicutes, Bacteroidetes, Proteobacteria, and Actinobacteria, dominated the Pakistani gut microbiome community comprising, on average, ~96% of the total bacteria load (Figure 2A, Table S2). There were, however, large fluctuations in the individual proportions of each of these core phyla across individuals (Figure 2A), which is not surprising since individuals typically follow a continuum rather than clustering into discrete groups when compared across a particular body site (Jeffery et al., 2012; Knights et al., 2014). Compared to the uBiome healthy cohort, however, both Pakistani males and females harbored lower mean proportions of Firmicutes (40.1% and 51.9% vs. 60.1% in uBiome) and Bacteroidetes (21.18% and 27.24% vs. 31.06%) but higher mean proportions of Proteobacteria (25.06% and 8.29% vs. 3.83%) and Actinobacteria (9.31% and 7.99% vs. 4.1%) (Figure 2B, Table S2). While, mean proportions of all four phyla were different from uBiome healthy individuals for both Pakistani men and women, women again appeared to more closely resemble the numbers in the uBiome cohort (Table S2).

### High Levels of Proteobacteria in Pakistani Male Samples Could Be of Concern

**Figure 3:**
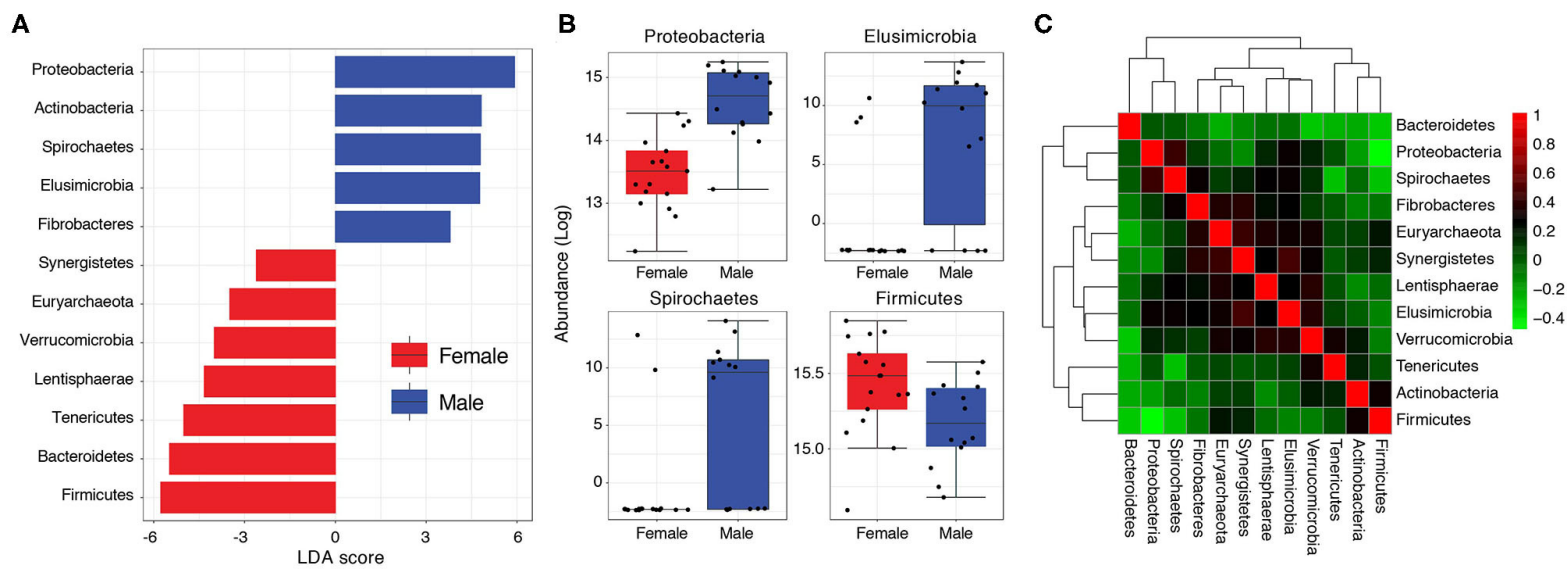
**(A)** Bar graph showing LDA scores of significant features (phyla) characterizing Pakistani male and female gut samples. **(B)** Boxplots comparing phyla with significant differential abundance in males and females (Wilcoxon rank-sum test, *FDR* < 0.05). **(C)** A heat-map visualizing how phyla abundances in the Pakistani gut correlate to each other (Kendall's τ).

The higher load of Proteobacteria in Pakistani individuals relative to the uBiome cohort (mean relative abundance = 15.86% vs. 3.83%) could be of concern. Proteobacteria levels >13% have previously been associated with metabolic disorders, inflammation and cancer, and considered a diagnostic marker for microbiota dysbiosis and disease (Shin et al., 2015). Proteobacteria load was especially very high in Pakistani men ranging from 5.53 to 41.84% (mean = 25.06%) compared to 2.06–18.53% (mean = 8.29%) in women (Table S2). In other words, even the minimum Proteobacteria load detected in any Pakistani male (5.53%) was still higher than the average Proteobacteria load (4.5%) typically expected in healthy individuals (Shin et al., 2015). A differential abundance analysis followed by linear discriminant analysis (LDA) confirmed these gender-wise differences. Proteobacteria, Actinobacteria, Spirochaetes, Elusimicrobioa, and Fibrobacteres characterized male samples (LDA >3) whereas Firmicutes, Bacteroidetes, Tenericutes, Lentisphaerae, Verrucomicrobia, Euryarchaeota, and Synergistetes characterized the female samples (Figure 3A). Relative abundances of Proteobacteria, Spirochaetes and Elusimicrobia were significantly higher in males relative to females (*FDR* < 0.05) whereas women had a relatively higher load of Firmicutes to males, albeit with marginal significance (*P* = 0.03, *FDR* = 0.1) (Figure 3B). Abundance of Proteobacteria was moderately correlated with Spirochaetes (Kendall's τ = 0.42) and negatively correlated with Firmicutes (τ = -0.49) (Figure 3C, see Table S3 for correlation scores).

### Pakistani Gut Microbiome Is Skewed Toward Firmicutes

The median Firmicutes to Bacteroidetes ratio in Pakistani samples was 2.16:1 (males = 1.82:1, females = 2.27:1, Figure S2). In fact, 26/31 (84%) of sampled individuals had Firmicutes to Bacteroidetes ratio of >1 (shaded areas in Figure S2). Despite the fact that the Pakistani gut microbiome seemed skewed toward Firmicutes, both Pakistani men and women had relatively fewer Firmicutes and Bacteroidetes compared to the uBiome healthy dataset (Figure S2). Only 5/17 (29%) and 6/17 (35%) women had comparable or greater number of Firmicutes and Bacteroidetes relative to uBiome healthy individuals whereas only 1/14 men had comparable or greater number of Bacteroidetes relative to uBiome healthy individuals (Figure S2). This suggests that despite the relatively lower proportions of these two key phyla in the Pakistani gut, there was much greater imbalance and, in general, the Pakistani gut microbiome was skewed toward Firmicutes. In the past, high Firmicutes to Bacteroidetes ratio has been associated with obesity but recent findings have contradictory these views (Magne et al., 2020). The biological value of the reported skewness therefore needs further investigation.

### *Succinivibrio* Was Found in High Abundance in the Pakistani Male Gut Microbiota

*Succinivibrio* genus (Proteobacteria) was detected in 19/32 samples with a mean relative abundance of 12.21% (Table S4). It was especially more prevalent and abundant in males (14/14 samples and mean relative abundance = 23.46%) than females (5/17 samples and mean = 2.94%) (Table S4). Thus, it could be responsible for observed over-representation of Proteobacteria in Pakistani male samples. Beta-diversity using weighted UniFrac distance reasonably separated male and female samples (54.8% variability explained by two principal coordinates) and confirmed the enrichment of *Succinivibrio* in male samples (Figure S3A). *Succinivibrio*, along with *Lachnospira* (Firmicutes), *Elusimicrobium* (Elusimicrobia), and *Phascolarctobacterium* (Firmicutes) had significant differential abundance in males and females (Figure S3B, *FDR* < 0.05). Except *Lachnospira*, rest were significantly over-represented in males (Figure S3B). In Pakistani samples, *Succinivibrio* was positively correlated with *Phascolarctobacter* (τ = 0.55, Table S5). *Succinivibrio* are typically over-represented in cattle switched from high-fiber to high-starch diet (Petri et al., 2013) and are integral members of the honey bee gut microbiota, which also relies on starch (Mattila et al., 2012). Thus, they are likely associated with starch metabolism (O'Herrin and Kenealy, 1993). An LDA determined 25 most significant features (genera) and their effect sizes (relevance) in categorizing males and females (Figure S3C). Along with *Succinivibrio, Elusimicrobium, Thalassospira, Phascolarctobacterium*, and *Varibaculum* characterized male samples with LDA scores of >|3| whereas *Faecalibacterium, Subdoligranulum, Collinsella, Alistipes, Lactobacillus, Clostridium, Dorea* (and several others) characterized female samples with LDA score >|3| (Table S6 for all features).

### A Coarse-Grained Snapshot of the Pakistani Gut Microbiome

The uBiome dataset indicated that *Faecalibacterium, Bacteroides, Roseburia*, and *Blautia* comprised >50% of total bacterial genera in the uBiome healthy cohort (Table S2). In turn, 17 different genera (detected in all 31 gut samples) collectively comprised the ~50% threshold in Pakistani samples (excluding *Succinivibrio* that was absent in many female samples) (Table S2). These genera included notable inhabitants of the human gut including *Faecalibacterium, Bacteroides, Roseburia, Blautia*, and *Bifidobacterium*. Interestingly all, except *Bifidobacterium*, were under-represented in the Pakistani samples relative to the uBiome dataset and once again Pakistani women had relatively higher mean abundances compared to Pakistani men (Table S2).

We detected high prevalence and abundance of *Bifidobacterium* (31/31 samples) and *Lactobacillus* (29/31) in the Pakistani gut samples (Table S4). *Bifidobacterium* are amongst the most well-studied microorganisms in the human gut (Schell et al., 2002). They are especially abundant in infants where they help digest indigestible oligosaccharides and fiber from breast milk and may protect gut from inflammation (Schell et al., 2002). Due to their usefulness, many *Bifidobacterium* species are sold as over-the-counter probiotics. *Bifidobacterium* comprised 4.21% of the total community (males = 3.69% and females = 4.65%) relative to 2.08% in uBiome dataset (Table S4). Similarly, *Lactobacillus* is also commercially sold as probiotics and was detected in most Pakistani samples, albeit with mean relative abundance of <1% (Table S4). Their wide presence could be due to the fact that milk-based products and fermented foods (e.g., yogurt) are very popular in the Pakistani diet, especially in Punjab. Moreover, infants are typically breastfed for up to 2 years for religious reasons throughout Pakistan (20/29 of our survey respondents, Table S1). Previously, an association between *Bifidobacterium* and milk-based diets has been established (Sela and Mills, 2010).

The most notable absentee from the majority of Pakistani samples was *Akkermansia* (Verrucomicrobia) that has been linked to many health benefits (Cani and de Vos, 2017; Naito et al., 2018) but also colon related diseases and infections in the absence of sufficient amount of dietary fiber (Desai et al., 2016). *Akkermansia* was one of the low-count and low-variance features removed during our analysis (see section Materials and Methods). In the original pre-filtered dataset, *Akkermansia* was detected in 2/32 samples at very negligible amounts (<0.06%) and in one sample (ISB-18) at 2.76% (data not shown).

### The Composition of the Oral Microbiome

**Figure 4:**
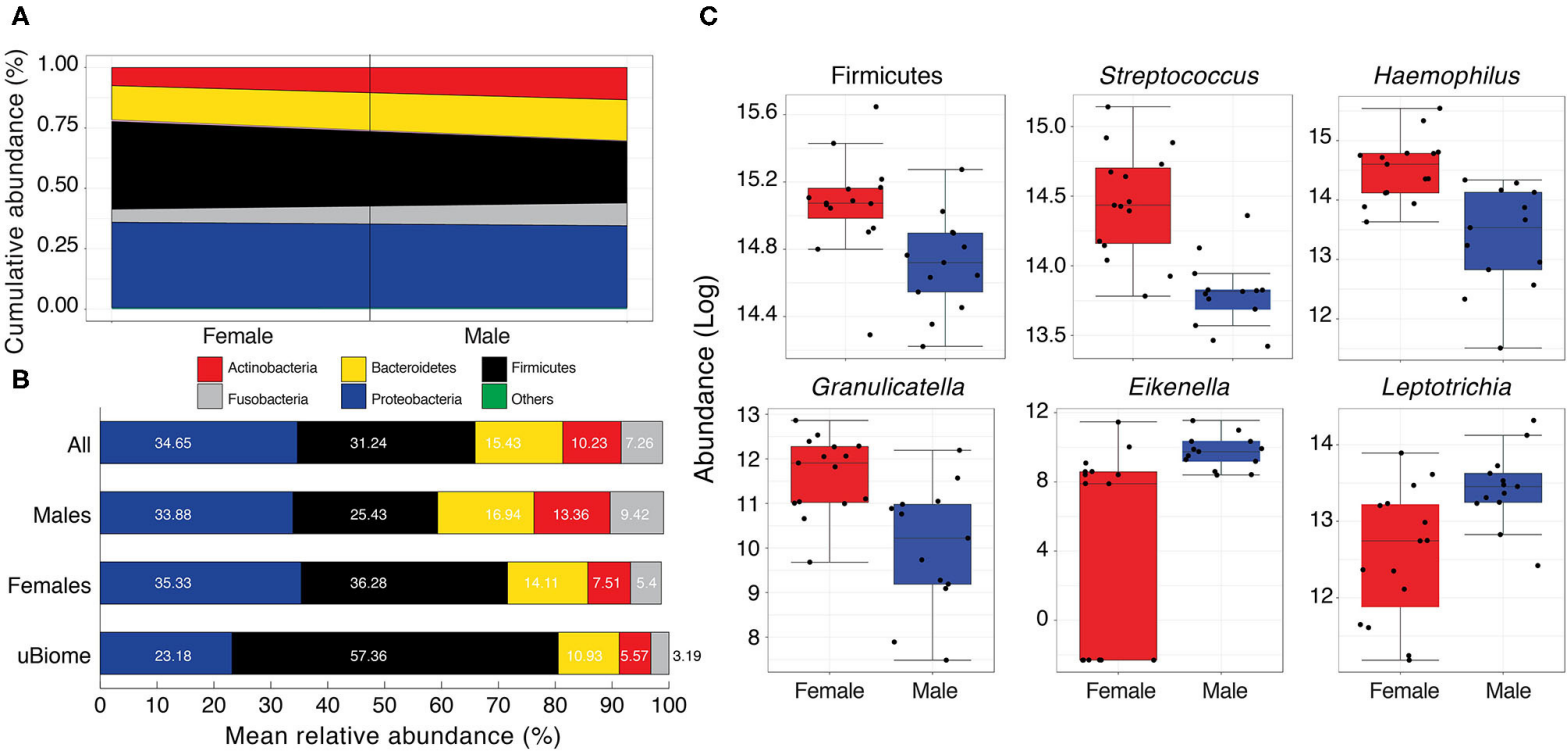
**(A)** Cumulative abundance (%) of major phyla detected in the Pakistani oral samples. Black line separates male and female samples. *Others* category includes Spirochaetes, *Cand*. Saccharibacteria, and Synergistetes (see Table S7 for actual values) **(B)** Comparison of mean relative abundance (%) of major phyla detected in the oral cavity of Pakistani males and females vs. uBiome dataset. Numbers on bars indicate actual percentages. **(C)** Boxplots comparing phyla and genera with significant differential abundance in males and females (Wilcoxon rank-sum test, *FDR* < 0.05).

The Pakistani oral microbiome (*n* = 28 samples) was dominated by five phyla constituting ~99% of the total microbial community (Figure 4A and Table S2). These included Proteobacteria, Firmicutes, Bacteroidetes, Actinobacteria, and Fusobacteria. *Cand*. Saccharibacteria (formerly TM7), Spirochaetes, and Synergistes were also detected, albeit in minor amounts (<2% cumulative) (Table S7). Once again, gender appeared to be the strongest determinant of microbiome variability among the Pakistani individuals. Similar to the gut microbiota profile, Firmicutes were significantly more abundant in women (36.28% vs. 25.43%, Figure 4). Compared to the uBiome dataset, Pakistani individuals were over-represented in all bacterial phyla except Firmicutes (31.24% vs. 57.36%, Figure 4 and Table S2).

Following quality control and filtering (see section Materials and Methods), 58 different genera were detected in the oral samples (Table S8). Among these, 18 were detected in all samples and included notable inhabitants of the oral cavity such as *Streptococcus, Neisseria, Haemophilus, Veillonella, Prevotella*, and *Gemella* (Table S2). While the mean relative abundances of these genera varied considerably among males and females, five genera (*Streptococcus* [Firmicutes], *Haemophilus* [Proteobacteria], *Granulicatella* [Firmicutes], *Eikenella* [Proteobacteria], and *Leptotrichia* [Fusobacteria]) had significant differential abundance in females and males (Figure 4B and Table S9). Of these, *Streptococcus, Haemophilus*, and *Granulicatella* were significantly more abundant in females vs. males (*FDR* < 0.05) while *Eikenella* and *Leptotrichia* were significantly more abundant in males vs. females (Figure 4C). *Haemophilus* and *Streptococcus* are commensal to the human body (Dewhirst et al., 2010) although some strains can be opportunistic pathogens (Tikhomirova and Kidd, 2013). Both were moderately correlated with *Gemella* (τ > 0.43, Table S10), which along with *Neisseria* and *Veillonella* are considered cornerstones of good oral hygiene. Some *Neisseria* subtypes have even been explored as possible probiotics to stave off tooth decay. Similar to *Bifidobacterium* and *Lactobacillus* in the human gut, *Neisseria* constituted a very good load in the Pakistani oral microbial community (mean relative abundance = 11.09% vs. 7.12% in the uBiome dataset, Table S2). *Neisseria* were moderately correlated with *Eikenlla* (τ = 0.44).

## Discussion

In this study, we performed 16S rRNA gene sequencing of the gut and oral microbial communities in 32 urban Pakistani adults (61 total samples). These individuals were “cherry-picked” to cover the maximum possible geographic and ethnic diversity of Pakistan in the early phases of the project so that we could design more improved and better-informed sampling studies in the future. Initially, we compared the Pakistani gut microbiota profiles against the uBiome reference dataset using the crude wellness match measure. Wellness match, as defined by uBiome Explorer, is the similarity/overlap (%) in the gut microbiome composition between the samples of interest and carefully selected individuals who self-reported to be in excellent medical condition and gave prior consent to participate and share their data for research projects. These data were available to both commercial users and uBiome grant awardees prior to uBiome closure in 2019. This measure, however, has no bearing on any person's health since it is only based on overlap in the 16S microbiota profiles. Moreover, the two datasets have large geographic differences (i.e., Pakistani vs. western populations). Over such large scales, lifestyle differences can easily be confounded by geography. The measure however resolved gender-wise differences within the Pakistani gut microbiota, which we explored further.

We observed that Pakistani women had significantly more Firmicutes and men had significantly more Proteobacteria. The high load of Proteobacteria in gut (typically >13%) has previously been associated with diseases and microbiota dysbiosis and could be a cause for concern (Shin et al., 2015). High load of Proteobacteria can also result from contamination or bacterial overgrowth during shipment of samples (Amir et al., 2017). Although, we strictly instructed and guided study participants to wash their hands and use sterile wipes/swabs for sampling, overgrowth or bloom of proteobacteria during transit cannot be ruled out, especially considering the small sample size in the pilot study. However, contamination should commonly affect both males and females, which we did not observe.

The observed gender-wise differences are interesting and could be due to many reasons. For example, we faced significant challenges in recruiting participants of normal BMI from urban areas. Roughly, 90% of the interviewed participants were either overweight or obese and were thus excluded. In observation, Pakistani men in urban regions are unconscious about their calorie intake and body weight and shape and do not exercise regularly. In contrast, Pakistani women are under social pressure to maintain body weight and shape, especially before marriage, and, in observation, consume fewer portions of the same food relative to Pakistani males. This could partially explain observed gender-based observed differences in microbiome profiles, especially since the majority of our participants were young and unmarried (24/32, Table S1). A time series monitoring of microbiota levels in Pakistani men and women from wider age brackets is therefore warranted to evaluate the robustness of our preliminary findings.

We now highlight additional challenges and limitations of the present analysis. First, antibiotic (mis)-use is frequent throughout Pakistan, which is now number three in the low or middle-income countries in antibiotic consumption (Klein et al., 2018). The majority of participants initially interviewed had consumed antibiotics in the past 6 months and we relaxed the criteria to 3-months as an afterthought (see Materials and Methods). Second, we only focused on bacteria (and to some extent archaea) via 16S rRNA gene sequencing and did not explore viruses and fungi (the mycobiome), the other big players in the human microbiota. Third, a major issue is of small sample size and distribution (mostly restricted to working and higher socioeconomic class). The present analysis therefore does not fully capture the full diversity of Pakistan. Fourth, comparisons between ethnicities and geographies are tentative given the small sample size and high heterogeneity between these groups. Time-series monitoring over a larger distribution and greater number of individuals is now the next step. Fifth, we collected data about the long-term dietary habits of study participants (Table S1) but not what they consumed immediately or 24 h prior to sampling. This missing information can potentially influence our findings since gut microbiota composition and diversity can be highly sensitive to short-term dietary changes (David et al., 2013). Finally, 16S based descriptive studies do not establish cause for any health-related matter and are merely descriptive. The value of the present study is in providing a useful snapshot of microbiome profile from a relatively undersampled population, which can hopefully influence and improve the design of future microbiome studies in the country.

## Data Availability Statement

The original contributions presented in the study are included in the article/Supplementary Material, further inquiries can be directed to the corresponding author/s.

## Ethics Statement

The studies involving human participants were reviewed and approved by COMSATS Ethics Review Board. The patients/participants provided written informed consent to participate in this study.

## Author Contributions

AN conceptualized the study. All authors contributed in the study design, conduct, and sample collection. uBiome performed DNA sequencing. MB and AJ performed downstream bioinformatics analysis. AN and MB wrote the draft. All authors contributed in the preparation and finalization of the manuscript.

## Conflict of Interest

The authors declare that the research was conducted in the absence of any commercial or financial relationships that could be construed as a potential conflict of interest.
