# Supplementary material for: Initial Sequencing and Characterization of the Gastrointestinal and Oral Microbiota in Urban Pakistani Adults
Source: Front Cell Infect Microbiol. 2020 Aug 7;10:409. doi: 10.3389/fcimb.2020.00409 (PMC7428047; doi:10.3389/fcimb.2020.00409)
Supplement: Supplementary file 11 [file Data_Sheet_1.docx]

Supplementary Material

Initial Sequencing and Characterization of the Gastrointestinal and Oral Microbiota in Urban Pakistani Adults

# Supplementary Data

Quality-controlled FASTQ files of 16S rRNA gene sequencing in gut and oral samples reported in this study have been deposited to figshare (10.6084/m9.figshare.7093565).

# Supplementary Figures and Tables

**2.1 Supplementary Figures**

**
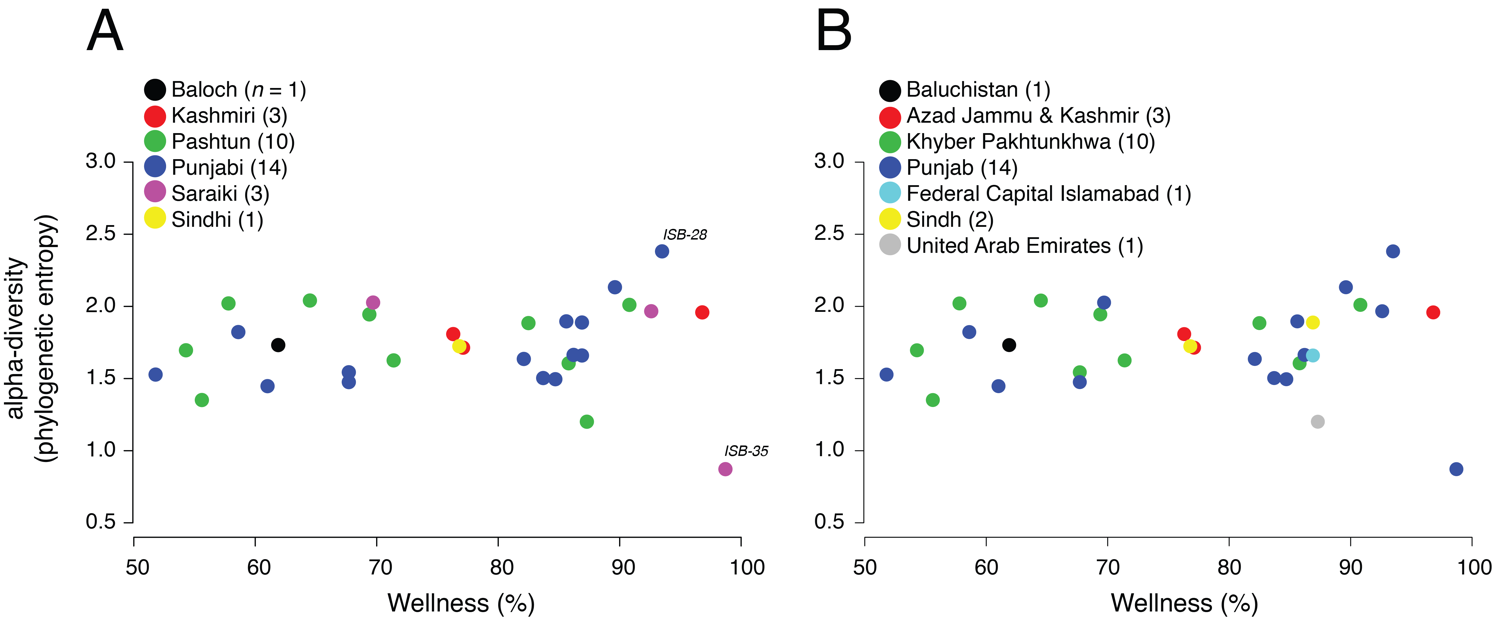
**

**Figure S1.** A scatter-plot displaying the relationship between wellness (%) and alpha-diversity (phylogenetic entropy) for ethnicities **(A)** and geographies **(B)**. AJK, Azad Jammu & Kashmir; KPK, Khyber Pakhtunkhwa.


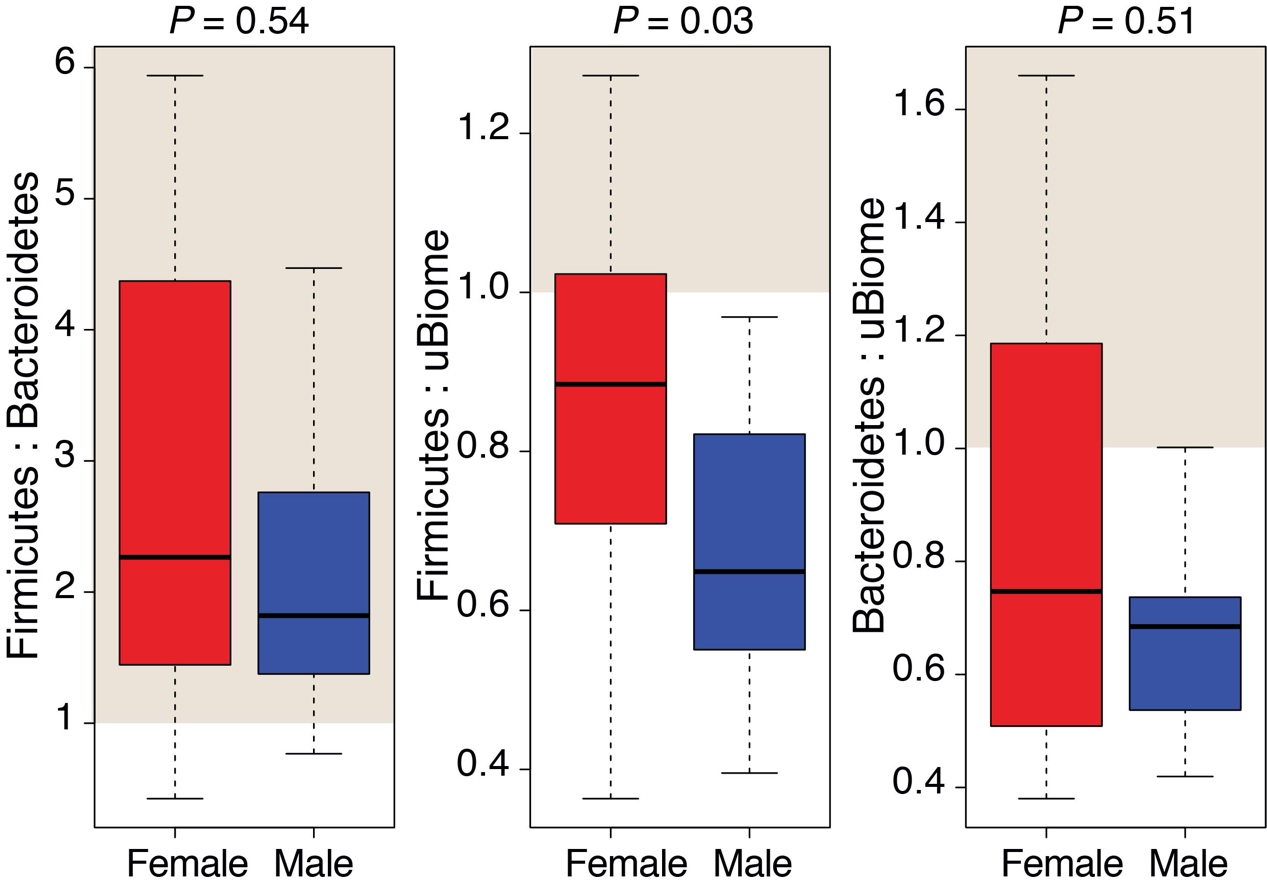


**Figure S2.** Firmicutes to Bacteroidetes ratio in Pakistani male and female gut samples and their comparison to the uBiome dataset. Shaded areas indicate samples where ratio exceeds 1. Statistical significance evaluated by two-tailed Wilcoxon rank sum test (*P* < 0.05).

**
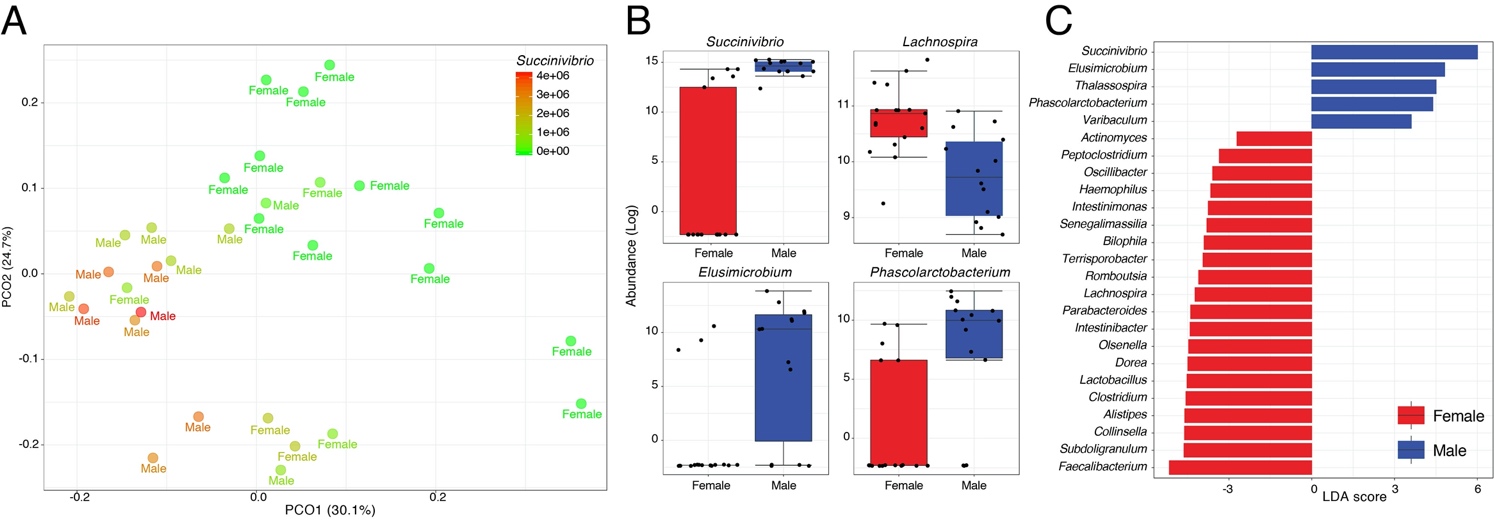
**

**Figure S3. (A)** A two-dimensional PCoA plot evaluates dissimilarity (Weighted UniFrac) between male and female samples. Samples are colored by *Succinivibrio* abundance. **(B)** Boxplots comparing genera with significant differential abundance in males and females (Wilcoxon rank-sum test, *FDR* < 0.05). **(C)** Bar graph showing LDA scores of top 25 significant features (genera).

**2.2 Supplementary Tables**

**Table S1.** Detailed characteristics of shortlisted study participants and their responses to health and dietary questionnaire.

**Table S2.** Mean, minimum (*min*), and maximum (*max*) relative abundance of major phyla and genera detected in gut and oral samples and their comparison to the uBiome dataset. uBiome numbers as reported on uBiome Explorer in July 2018.

**Table S3.** Kendall’s rank (τ) abundance correlations for microbial phyla detected in Pakistani gut samples.

**Table S4.** Mean, minimum (*min*), and maximum (*max*) relative abundance of microbial genera detected in all, male, and female Pakistani gut samples. *Succinivibrio* is highlighted.

**Table S5.** Kendall’s rank (τ) abundance correlations for microbial genera detected in Pakistani gut samples.

**Table S6.** Differential abundance analysis of microbial genera across gender in Pakistani gut samples. Statistically significant differences (*FDR* < 0.05) are highlighted.

**Table S7.** Mean, minimum (*min*), and maximum (*max*) relative abundance of microbial phyla detected in all, male, and female Pakistani oral samples.

**Table S8.** Mean, minimum (*min*), and maximum (*max*) relative abundance of microbial genera detected in all, male, and female Pakistani oral samples.

**Table S9.** Differential abundance analysis of microbial genera across gender in Pakistani oral samples. Statistically significant differences (*FDR* < 0.05) are highlighted.

**Table S10.** Kendall’s rank (τ) abundance correlations for microbial genera detected in Pakistani oral samples.
